# Epidemiological Trends, Inter-Cancer Correlations, and Incidence Projections for 61 Cancer Types in Korea, 1999–2028: A Nationwide Population-Based Study

**DOI:** 10.3390/cancers18142341

**Published:** 2026-07-20

**Authors:** Hyeran Jung, Minsun Jung

**Affiliations:** Department of Pathology, Yonsei University College of Medicine, Seoul 03722, Republic of Korea; phdgrace@yuhs.ac

**Keywords:** cancer registry, age-standardized rate, time series forecasting, Holt–Winters, ARIMA, hierarchical regression, multicollinearity, ecological study, annual percent change, five-year prevalence

## Abstract

**Background/Objectives:** Korea has undergone rapid epidemiological transitions in cancer incidence over the past two decades. Using a 25-year nationwide dataset (1999–2023), we characterize long-term trends for 61 cancer types, examine inter-cancer correlations, and forecast incidence to 2028. **Methods:** Annual incidence counts, crude rates, and age-standardized incidence rates (ASIRs) stratified by sex were obtained from the Korea Central Cancer Registry (KCCR) via the Korean Statistical Information Service (KOSIS). Annual percent change (APC) was estimated using log-linear regression. Pearson correlation coefficients were computed among cancer-specific ASIRs, with false-discovery-rate (FDR) correction for multiple comparisons. Multiple and hierarchical regression evaluated the statistical association of individual cancer types with the overall cancer rate, and variance inflation factors (VIFs) were used to quantify multicollinearity. Time series forecasting used damped Holt–Winters exponential smoothing; forecast accuracy was assessed with rolling-origin cross-validation (RMSE, MAE, MAPE) and benchmarked against ARIMA. A sensitivity analysis excluding the pandemic years (2020–2021) tested the robustness of trend estimates. Five-year prevalence data (2007–2023) were analyzed from the KCCR prevalence module. **Results:** Total cancer incidence increased from 101,854 in 1999 to 288,613 in 2023, a 183% increase. The overall ASIR rose from 402.7 to 522.9 per 100,000 (2020 standard population). The three fastest-growing cancers were thyroid (APC +7.56%, *p* < 0.001), prostate (+6.98%, *p* < 0.001), and breast (+5.03%, *p* < 0.001). Stomach (APC −2.20%) and liver (−2.90%) cancers showed significant declines. Hierarchical regression showed that adding thyroid, breast, and prostate to lung and stomach increased explained variance from R^2^ = 0.449 to 0.997; however, high VIF values (up to ~263) indicate substantial multicollinearity and compositional dependence, so these coefficients should not be read as independent causal contributions. Holt–Winters and ARIMA produced comparable accuracy (mean MAPE 4.6% vs. 4.7%). The five-year cancer prevalence pool reached 1,035,107 in 2023. Forecasting projects a total incidence of approximately 319,000 by 2028. **Conclusions:** Korean cancer epidemiology is undergoing a transition from infection-related cancers toward hormone-sensitive and screening-detectable malignancies. These findings support strategic resource allocation for high-growth cancers while maintaining vigilance over rising pancreatic and other emerging cancers.

## Simple Summary

Cancer remains a leading public health burden in Korea. Using 25 years of national cancer registry data covering 61 cancer types (1999–2023), we describe incidence trends, analyze statistical correlations among cancer types, and project future incidence to 2028 using Holt–Winters exponential smoothing. Our analysis reveals marked epidemiological transitions: thyroid, breast, and prostate cancers have shown rapid increases, whereas stomach and liver cancers continue to decline. Hierarchical regression identifies thyroid, breast, and prostate cancer as the strongest population-level statistical correlates of overall cancer incidence. Because these analyses are ecological and the candidate predictors are components of the overall rate, we interpret them as descriptive associations rather than causal or independent drivers. Projections indicate that overall cancer incidence will continue to rise through 2028. These findings provide a comprehensive, data-driven foundation for national cancer control planning in Korea.

## 1. Introduction

Cancer is a leading cause of death worldwide and represents one of the most pressing public health challenges of the twenty-first century. According to GLOBOCAN 2022, approximately 20 million new cancer cases were diagnosed globally and 9.7 million cancer deaths occurred, underscoring the enormous scale of the oncological burden [1,2,3,4]. In Korea, the cancer burden has grown substantially in parallel with rapid socioeconomic development, population aging, and lifestyle transitions since the late twentieth century.

The Korea Central Cancer Registry (KCCR), established in 1980 and operating under the auspices of the Ministry of Health and Welfare, provides high-quality, population-level cancer surveillance data. Annual cancer statistics derived from this registry have documented a sustained rise in overall cancer incidence, from approximately 100,000 new cases per year in the late 1990s to more than 280,000 per year in the early 2020s [5]. Beyond the aggregate burden, however, the epidemiological landscape has been highly heterogeneous across cancer types; some malignancies—notably thyroid, prostate, and female breast cancers—have experienced dramatic increases, whereas others, including stomach and liver cancers, have undergone substantial declines [2,6,7].

Understanding these divergent trends and the inter-relationships among cancer types is of considerable public health importance. Cancers sharing common etiological drivers—such as obesity, reproductive factors, or viral hepatitis—may exhibit correlated temporal trends, and the magnitude of these correlations can inform integrated surveillance and prevention strategies. Moreover, the ability to project future cancer incidence provides an evidence base for healthcare capacity planning and resource allocation.

Previous studies have characterized cancer trends in Korea over shorter observation windows or focused on specific cancer sites [7,8,9]. Comprehensive analyses covering all 61 internationally classified cancer types across 25 years (1999–2023), incorporating correlation analyses among cancer types and multi-horizon forecasting, remain limited. Furthermore, the addition of 5-year prevalence data provides a fuller picture of the survivorship burden, which has not been systematically integrated with incidence trend analysis in prior Korean reports.

The present study addresses these gaps by (1) describing trends in incidence and age-standardized incidence rates for 61 cancer types from 1999 to 2023, stratified by sex; (2) quantifying inter-cancer correlations using Pearson correlation analysis; (3) examining which cancer-type rates are most strongly associated with the overall cancer rate through hierarchical regression, while explicitly evaluating multicollinearity and compositional dependence; and (4) generating cross-validated Holt–Winters exponential smoothing forecasts, benchmarked against ARIMA, for the period 2024–2028. This analysis is intended to inform national cancer control policy and support evidence-based healthcare planning.

## 2. Materials and Methods

### 2.1. Data Sources

Cancer incidence data were obtained from the Korea Central Cancer Registry (KCCR), publicly available through the Korean Statistical Information Service (KOSIS; https://kosis.kr), administered by the Ministry of Health and Welfare (MOHW Table ID: DT_117N_A00025) [10,11]. Data covered all 61 International Classification of Diseases for Oncology (ICD-O) cancer types, stratified by sex (total, male, female), for the years 1999–2023. Variables included annual new case counts (N), relative frequency (%), crude incidence rate (per 100,000), and age-standardized incidence rate (ASIR; per 100,000). ASIRs were computed using Korea’s 2020 mid-year resident registration census as the standard population. In December 2025, updated 2023 cancer registration statistics were released, and all 1999–2022 incidence data were retrospectively revised accordingly; the present analysis uses this most recent revision throughout [5,12].

Five-year cancer prevalence data (2007–2023) were obtained from the KCCR prevalence module (KOSIS Table ID: DT_117N_A00121), providing five-year prevalent case counts, relative frequency, crude prevalence rate, and age-standardized prevalence rate (ASPR; per 100,000, defined as the five-year prevalent case count standardized to the 2020 Korean standard population) by sex and cancer type. All rate-based comparisons in this study emphasize age-standardized rates (ASIR and ASPR), because crude rates are confounded by the marked aging of the Korean population over the study period and are therefore less suitable for temporal comparison.

### 2.2. Statistical Analysis

All statistical analyses were performed using Python (version 3.12; https://www.python.org) with NumPy (version 1.26; https://numpy.org), pandas (version 2.2; https://pandas.pydata.org), SciPy (version 1.12; https://scipy.org), statsmodels (version 0.14; https://www.statsmodels.org), and Matplotlib (version 3.8; https://matplotlib.org).

**Annual Percent Change (APC).** To quantify the direction and magnitude of incidence trends for each cancer type, we fitted log-linear regression models of the form log(ASIR) = α + β × Year. The APC was calculated as APC (%) = (e^β^ − 1) × 100. Statistical significance was assessed using two-sided *t*-tests with α = 0.05. We report a single APC across 1999–2023 as a parsimonious summary of the average long-term trend. We acknowledge that Korean cancer incidence has experienced well-documented structural changes—most notably the thyroid cancer surge and subsequent plateau around 2011–2016 and the COVID-19 disruption in 2020—that a single log-linear slope cannot capture. Joinpoint regression [13] is the standard tool for detecting such change-points and has been used extensively by the KCCR; we did not re-estimate joinpoints here because national joinpoint/AAPC trends are already published in the official statistics [5,6], and our objective was a harmonized cross-cancer comparison and forecasting framework rather than change-point detection. The single-slope APC should therefore be read as a period-average descriptor, and the non-linear dynamics are addressed separately in the sex-stratified trend figures and the forecasting models.

**Correlation Analysis.** Pearson correlation coefficients were computed among ASIRs of all 61 cancer types over the 25-year period, using all available annual data points. Correlation matrices were visualized as heatmaps for the 20 cancer types with the highest mean ASIR. Pearson correlation (rather than a time series-specific measure) was chosen because our aim was descriptive—to summarize the degree of co-movement between the standardized long-term trajectories of cancer pairs—rather than to model lead–lag dynamics. Because all series share a common secular time trend, we emphasize that these coefficients partly reflect shared temporal dependence and do not imply shared biological mechanisms or individual-level associations (the ecological fallacy [14]). Given the large number of pairwise tests (1830 unique pairs among 61 cancers), *p*-values were adjusted using the Benjamini–Hochberg false-discovery-rate (FDR) procedure, and significance is reported after correction.

**Multiple and Hierarchical Regression.** To assess the statistical association of cancer-specific ASIRs with the overall cancer ASIR, we constructed a series of ordinary least squares (OLS) regression models. Hierarchical regression was conducted in three blocks: Block 1 included lung and stomach cancer (historically dominant cancers); Block 2 added thyroid, breast, and prostate cancer (rapidly increasing cancers); Block 3 included all top 10 cancers by 2023 incidence. The increment in explained variance (ΔR^2^) at each step was evaluated using F-tests, and Cohen’s f^2^ was reported as an effect-size measure for each increment. This analysis was framed as descriptive variance-partitioning, not causal modeling. Two important caveats apply and are quantified in the Results: (i) the overall ASIR is, by construction, a weighted sum that mathematically contains the component cancer rates used as predictors, so a very high R^2^ is expected and does not indicate explanatory insight (compositional dependence); and (ii) the predictors are strongly inter-correlated. We therefore computed variance inflation factors (VIF) for the Block-3 predictors and interpret the individual coefficients with corresponding caution rather than as independent contributions [15].

**Time Series Forecasting.** Annual incidence projections for 2024–2028 were generated using damped Holt–Winters exponential smoothing [16,17,18], implemented via the statsmodels ExponentialSmoothing function with additive trend and optimized damping. Because the series are annual with no within-year seasonality, only level and (damped) trend components were estimated; the smoothing parameters (α, β) and damping parameter (φ) were estimated by maximum likelihood and are reported per cancer in Table 5. Forecast uncertainty was expressed as 95% prediction intervals computed from the standard deviation of the in-sample one-step residuals, scaled by the square root of the forecast horizon (a standard approximation for exponential-smoothing point forecasts). Predictive accuracy was evaluated by rolling-origin (time-series) cross-validation—successively expanding the training window from 1999 and forecasting the next year—which respects temporal ordering and is more appropriate for forecasting than leave-one-out cross-validation; we report root mean squared error (RMSE), mean absolute error (MAE), and mean absolute percentage error (MAPE) [19]. As a benchmark, an ARIMA(1,1,1) model was fitted to each series under the identical rolling-origin scheme, and its accuracy is compared with Holt–Winters in Table 5 (see Section 3.5) [20,21]. All forecasts assume that the historical trend and age structure evolve smoothly; they do not incorporate exogenous future changes in screening policy, demographic shifts beyond those embedded in the historical series, or novel diagnostic technologies.

**Robustness to the COVID-19 disruption.** Because 2020 incidence fell sharply during pandemic-related screening interruptions, we repeated the APC estimation after excluding 2020–2021 and compared the resulting slopes with the full-period estimates (Table 6) to confirm that the reported long-term trends were not artefacts of the pandemic dip.

### 2.3. Ethical Approval

This study utilized publicly available, fully de-identified aggregate cancer registry statistics published by the Ministry of Health and Welfare of the Republic of Korea. No individual-level patient data were accessed or analyzed. Ethical review and approval were waived by the Institutional Review Board of Yonsei University (IRB No. 4-2026-0352; approval date: 7 May 2026). Informed consent was waived because the study used deidentified aggregate national statistics and did not involve direct contact with participants.

## 3. Results

### 3.1. Overall Cancer Incidence Trends (1999–2023)

**Figure 1:**
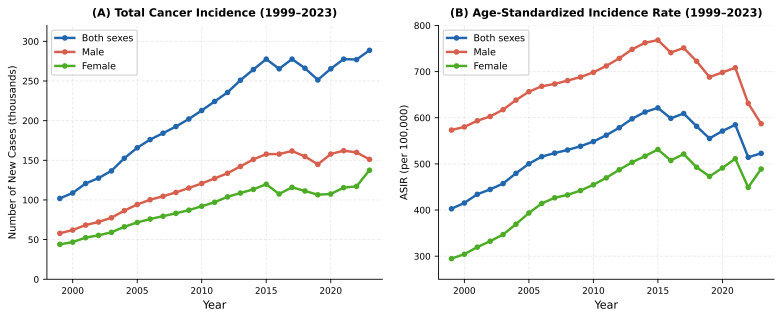
Total cancer incidence and age-standardized incidence rates (ASIR) in Korea, 1999–2023. (**A**) Annual new cancer cases by sex. (**B**) Age-standardized incidence rates (per 100,000; 2020 Korean standard population) by sex. A temporary decline is visible in 2020, discussed in Section 3.6.

Total cancer incidence in Korea increased from 101,854 new cases in 1999 to 288,613 in 2023, representing a 183.4% increase over 25 years (Figure 1). Male incidence grew from 57,883 to 151,126 cases (+161.1%), while female incidence grew from 43,971 to 137,487 (+212.7%). The overall crude incidence rate rose from 216.0 to 564.3 per 100,000. The ASIR (2020 standard population) increased from 402.7 to 522.9 per 100,000 for both sexes combined. Male ASIR rose from 573.3 to 587.0 per 100,000 (a modest increase reflecting population aging), while female ASIR increased from 294.7 to 488.9 per 100,000 (a 65.9% increase). A notable dip in total incidence was observed in 2020 (251,329 cases), which we interpret as most likely reflecting coronavirus disease (COVID-19)-related disruptions to cancer screening services (see Section 3.6), followed by a recovery to record levels by 2023.

**Figure 2:**
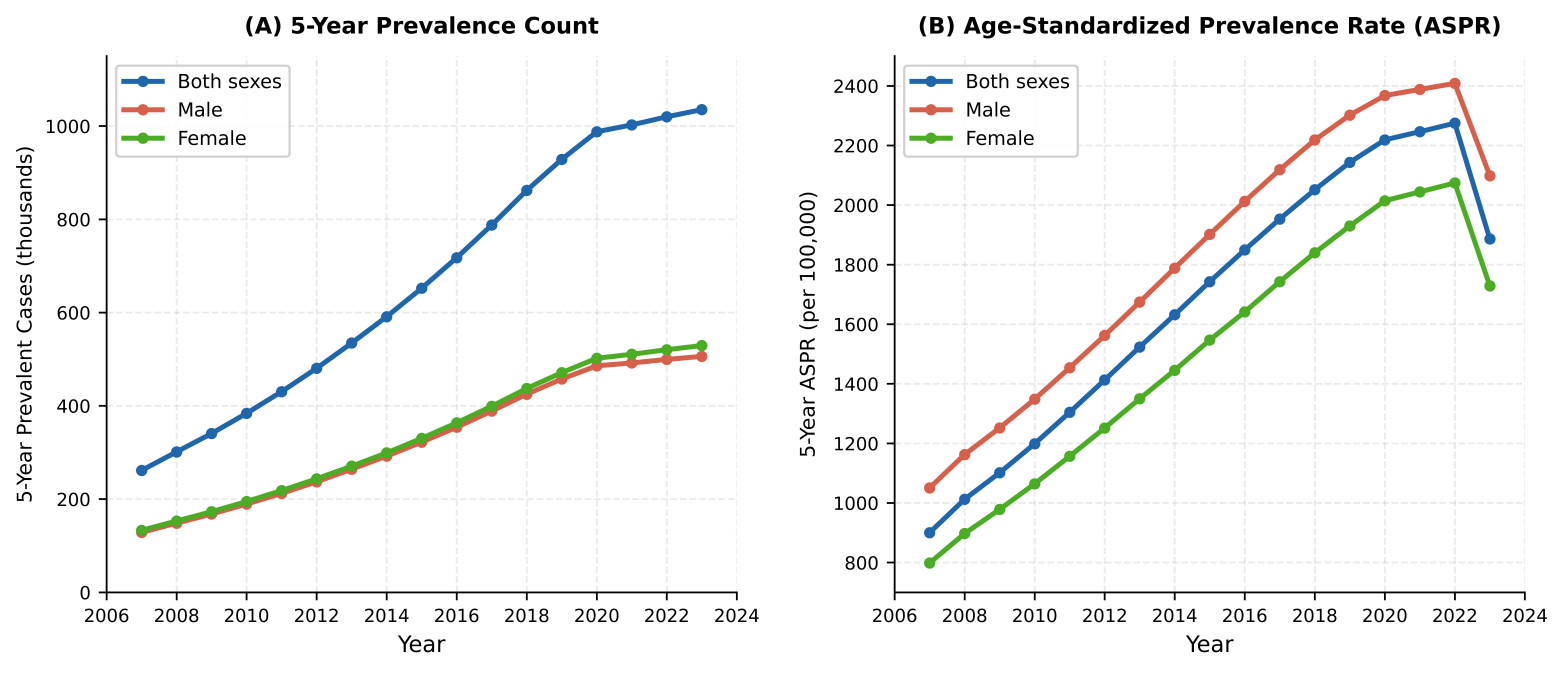
Five-year cancer prevalence in Korea, 2007–2023. (**A**) Annual five-year prevalent case counts by sex. (**B**) Age-standardized five-year prevalence rates (ASPR; per 100,000; 2020 Korean standard population) by sex.

The five-year cancer prevalence increased from 261,355 in 2007 to 1,035,107 in 2023, a 296% increase, reflecting improved survival and a growing survivorship population (Figure 2). The age-standardized five-year prevalence rate (ASPR) reached 1885.9 per 100,000 in 2023.

### 3.2. Cancer-Specific Trends and Annual Percent Change

**Figure 3:**
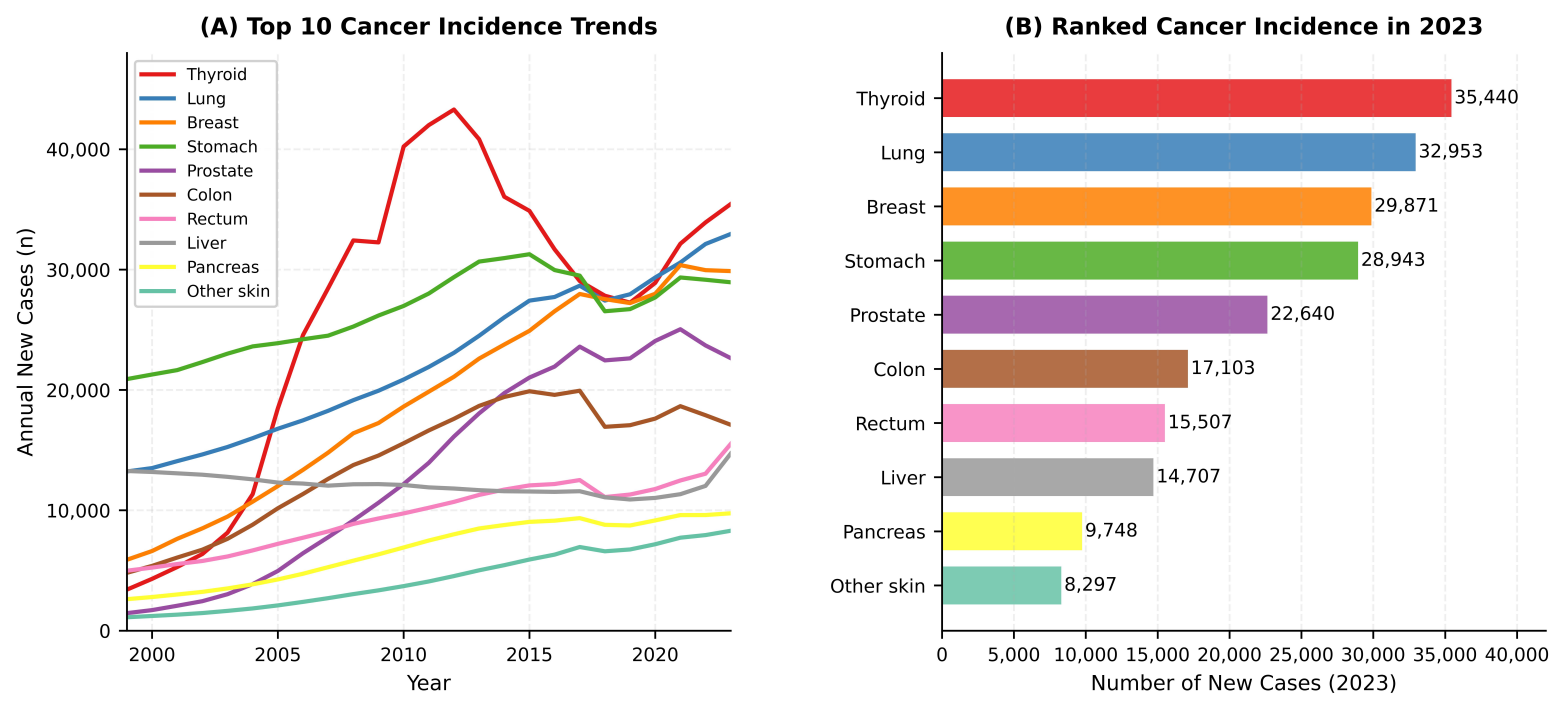
Top 10 cancers by incidence in Korea, 1999–2023. (**A**) Annual incidence trends for the 10 highest-incidence cancers. (**B**) Ranked cancer incidence in 2023, with total new case counts.

Detailed incidence data and APC estimates for the top 10 cancers are presented in Table 1. Among cancer types with the most rapid increases, thyroid cancer exhibited the highest APC (+7.56%, *p* < 0.001), rising from 3407 cases in 1999 to 35,440 in 2023—an approximately 10-fold increase. Prostate cancer also showed rapid growth (+6.98%, *p* < 0.001), increasing from 1454 to 22,640 cases. Breast cancer increased from 5888 to 29,871 cases (APC +5.03%, *p* < 0.001), making it the third most common cancer overall in 2023 (Figure 3). APC estimates for all 61 cancer types are provided in Supplementary Table S1.

**Figure 4:**
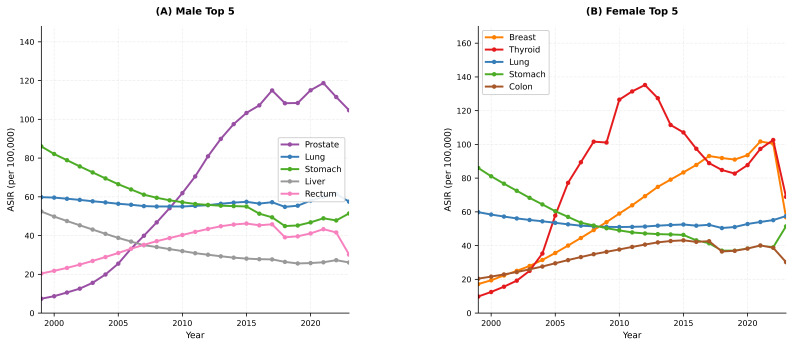
Sex-stratified age-standardized incidence rates for the five leading cancers among (**A**) males and (**B**) females in Korea, 1999–2023.

In contrast, stomach cancer—historically Korea’s most prevalent cancer—showed a significant downward ASIR trend (APC −2.20%, *p* < 0.001), with absolute cases rising modestly from 20,900 in 1999 to 28,943 in 2023 owing to population growth and aging. Liver cancer exhibited the steepest ASIR decline (APC −2.90%, *p* < 0.001). Lung cancer incidence showed no significant change in ASIR (APC −0.13%, *p* = 0.061), despite increasing absolute case counts. Pancreatic cancer showed a continuous significant rise (APC +1.65%, *p* < 0.001). Sex-stratified ASIR trends are shown in Figure 4.

### 3.3. Inter-Cancer Correlation Analysis

**Figure 5:**
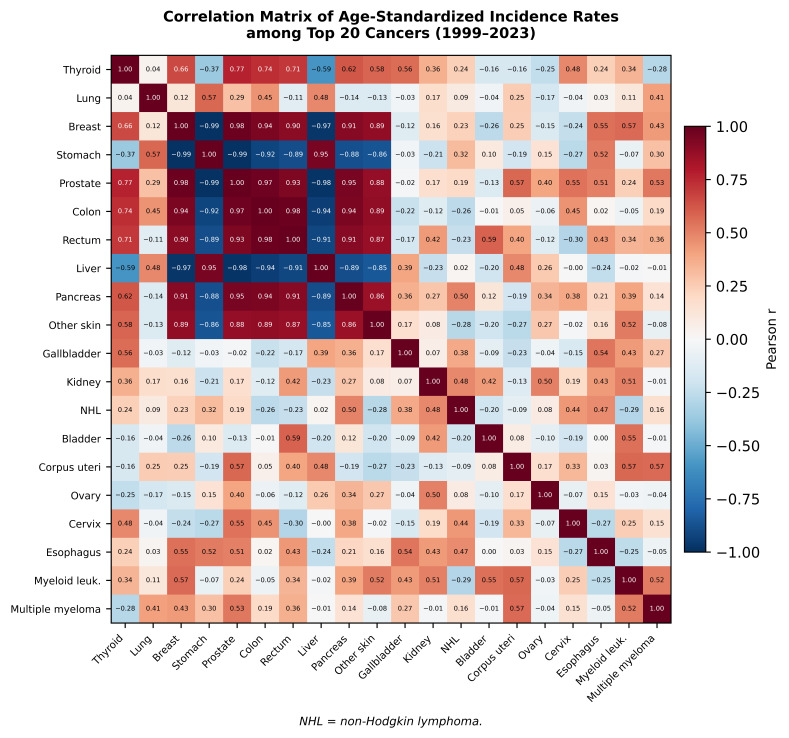
Pearson correlation matrix of age-standardized incidence rates among the top 20 cancers in Korea (1999–2023). Red indicates positive correlation; blue indicates negative correlation. Coefficients are displayed in each cell. NHL = non-Hodgkin lymphoma.

Pearson correlation analysis among ASIRs of the 20 cancers with the highest mean rates revealed substantial clustering of cancer trends (Figure 5). The strongest positive association was between breast and prostate cancer (r = 0.98). Thyroid cancer was moderately-to-strongly positively correlated with prostate (r = 0.77) and breast (r = 0.66) cancer. Conversely, stomach and liver cancers were strongly negatively correlated with breast, prostate, and pancreatic cancers (r ≈ −0.85 to −0.99), reflecting the divergent trajectories of infection-related versus hormone- and lifestyle-related malignancies; their negative correlations with thyroid cancer were weaker (stomach −0.37, liver −0.59), consistent with thyroid cancer’s distinctive non-monotonic trajectory. Lung cancer showed only weak-to-moderate correlations with other sites (e.g., lung–liver r = 0.48). After Benjamini–Hochberg FDR correction, the strong positive and negative associations noted above remained statistically significant, whereas several weaker pairwise correlations did not. Crucially, because every series shares a common secular time trend, these coefficients largely reflect shared temporal patterning; they should be read as descriptive co-movement at the population level and not as evidence of shared biological mechanisms or of associations at the individual level.

### 3.4. Multiple and Hierarchical Regression Analysis

Hierarchical regression quantified how strongly cancer-specific ASIRs are associated with the overall cancer ASIR (Table 2). Block 1 (lung + stomach) explained 44.9% of variance in total ASIR (R^2^ = 0.449, F = 8.98, *p* = 0.0014). Adding thyroid, breast, and prostate in Block 2 produced a large increase in explained variance (ΔR^2^ = 0.547; Cohen’s f^2^ = 161.4), reaching R^2^ = 0.997. Block 3 (all top 10 cancers) yielded marginal additional improvement (ΔR^2^ = 0.003, R^2^ = 0.9997). This near-perfect fit is expected rather than informative: the overall ASIR is a weighted composite that mathematically contains its component cancer rates, so the regression largely re-expresses an accounting identity (compositional dependence).

In the Block-3 model, several component rates were individually significant, but their coefficients are not interpretable as independent contributions because of severe multicollinearity: variance inflation factors ranged up to ~263 (Table 3), far exceeding conventional thresholds (VIF > 10). We therefore refrain from ranking cancers as “drivers” of the national burden on the basis of these coefficients and instead treat the analysis as a descriptive demonstration that the post-1999 rise in overall incidence is dominated by the co-movement of thyroid, breast, and prostate cancer.

### 3.5. Cancer Incidence Projections, 2024–2028

**Figure 6:**
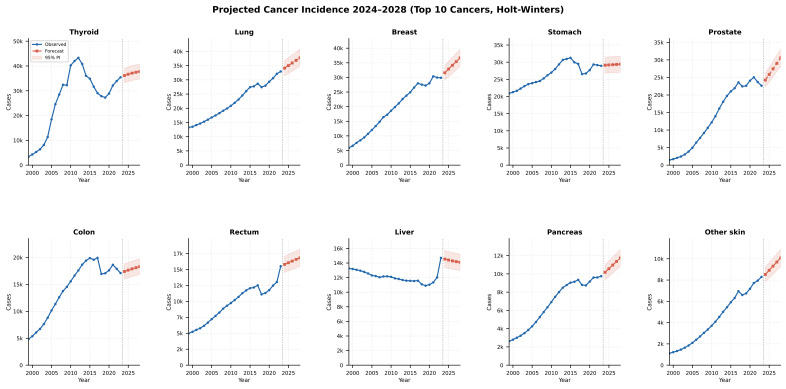
Damped Holt–Winters forecasts for the top 10 cancers in Korea, 2024–2028. Blue lines/circles denote observed incidence (1999–2023); red dashed lines/squares denote projected values; shaded areas are 95% prediction intervals.

Damped Holt–Winters forecasts are presented in Figure 6 and Table 4. For all cancers combined, total incidence is projected to rise steadily to approximately 295,000 in 2024, 301,000 in 2025, 307,000 in 2026, 313,000 in 2027, and 319,000 by 2028. Thyroid cancer is projected to reach 37,766 by 2028. Prostate cancer shows the steepest relative growth, projected to increase from 22,640 in 2023 to 30,593 in 2028. Breast cancer is projected to reach 36,702 by 2028.

**Figure 7:**
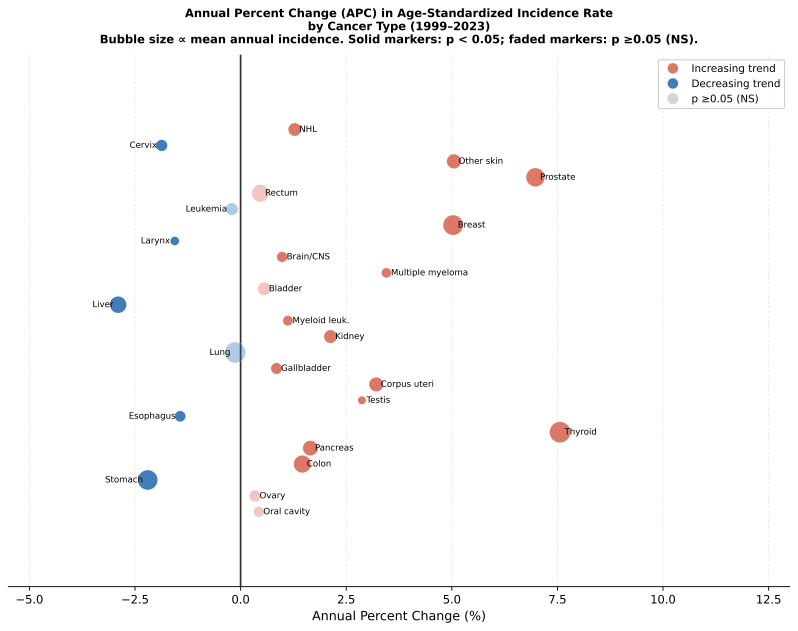
Annual percent change (APC) in age-standardized incidence rates by cancer type in Korea, 1999–2023. Red markers, increasing trends; blue markers, decreasing trends. Bubble size is proportional to mean annual incidence. Solid markers indicate statistically significant trends (*p* < 0.05); faded markers indicate non-significant trends.

Forecast validation is summarized in Table 5. Rolling-origin cross-validation showed good accuracy for most cancers (mean MAPE 4.6%). Holt–Winters and the ARIMA(1,1,1) benchmark performed comparably (mean MAPE 4.6% vs. 4.7%; mean RMSE 1272 vs. 1331 cases), supporting the adequacy of the parsimonious exponential-smoothing model; ARIMA was marginally better for stomach and prostate, and Holt–Winters for pancreas and other skin. Thyroid showed the largest errors under both models, reflecting its strongly non-linear historical trajectory (Figure 7).

### 3.6. Robustness to the COVID-19 Disruption

Total incidence fell to 251,329 cases in 2020 before rebounding, coinciding with documented pandemic-related reductions in national cancer screening participation [22]. To ensure that this transient dip did not distort long-term trend estimates, APCs were re-estimated after excluding 2020–2021 (Table 6, Section 3.6). Trend directions and magnitudes were essentially unchanged for all top 10 cancers (largest absolute change 0.62 percentage points, for thyroid), confirming that the reported trends are robust to the pandemic disruption.

## 4. Discussion

This study provides a comprehensive long-term analysis of cancer epidemiology in Korea, integrating 25 years of nationwide registry data for 61 cancer types with inter-cancer correlation analysis, hierarchical regression, and cross-validated incidence forecasting. Several findings merit discussion, which we frame throughout as hypotheses consistent with prior literature rather than as causal inferences derived from these aggregate data.

First, the magnitude of the overall increase in cancer incidence is striking. The near-tripling of annual new cancer cases between 1999 and 2023 reflects the combined effects of population growth, rapid aging, and—for several cancer types—genuine increases in age-standardized risk. The parallel increase in five-year prevalence to over one million persons underscores the growing survivorship burden on healthcare systems and social services.

Second, the differential trajectories across cancer types are consistent with an ongoing epidemiological transition. For thyroid cancer, the acceleration through approximately 2011 followed by a decline and partial recovery is widely attributed to changes in ultrasound-based screening intensity and the ensuing overdiagnosis controversy documented by Ahn et al. [23]; the 2015 guidance discouraging routine thyroid ultrasound screening plausibly contributed to the mid-decade decline. We emphasize that our ecological data cannot confirm these mechanisms directly. The rise in breast cancer incidence has been linked in prior individual-level studies to westernization of reproductive patterns and to population-level mammographic screening [24]. Prostate cancer growth is thought to reflect both PSA-testing diffusion and changes in diet and lifestyle. The decline in stomach and liver cancer is consistent with hepatitis B vaccination and antiviral therapy, reductions in Helicobacter pylori prevalence, dietary change, and the maturation of the national gastric cancer screening program [25,26,27]. For thyroid and prostate cancer in particular, a substantial component of the observed increase is likely attributable to detection and overdiagnosis rather than to a true rise in clinically important disease, a point now well recognized in the Korean and international literature [23,28]; this distinction is important for interpreting the forecasts, which project detected—not necessarily clinically significant—incidence.

Third, the hierarchical regression shows that the post-1999 rise in the overall rate is statistically dominated by the co-movement of thyroid, breast, and prostate cancer. However, as detailed in Section 3.4, the near-perfect model fit is a mathematical consequence of compositional dependence (the outcome contains its predictors) and the coefficients are confounded by extreme multicollinearity (VIF up to ~263). We therefore deliberately avoid describing any cancer as an independent “driver” of national burden on the basis of these coefficients, and we caution readers against such an interpretation. Likewise, the inter-cancer correlations should not be read as evidence of shared biological mechanisms; at the population level, cancers with any monotonic upward trend will correlate simply because they share a common time trend, and population-level correlations need not hold at the individual level (the ecological fallacy) [14].

Fourth, our forecasts project continued increases in total incidence, with prostate and pancreatic cancer showing the steepest relative growth. Holt–Winters and ARIMA yielded comparable out-of-sample accuracy, and the trend estimates were robust to exclusion of the pandemic years, which strengthens confidence in the descriptive projections. The projected continued rise in pancreatic cancer is of particular concern given its poor prognosis and the absence of an effective population screening tool [29]. These projections are broadly consistent with the National Cancer Center’s official Korean projections [6] and with model-based estimates for 2025 [6,30].

Fifth, our findings are relevant beyond Korea. The epidemiological transition observed here—declining infection-related cancers (stomach, liver) alongside rising hormone-sensitive and screening-detectable cancers (thyroid, breast, prostate)—mirrors patterns reported in other high-income East Asian populations such as Japan and, increasingly, urban China, and anticipates trajectories that other rapidly developing Asian countries may follow as they undergo similar demographic and lifestyle transitions [1,2]. Korea’s experience with thyroid cancer overdiagnosis, in particular, offers an internationally instructive cautionary case for health systems scaling up opportunistic screening. At the same time, the specific magnitudes reported here are shaped by Korea’s screening programs, diagnostic capacity, and population structure, so the quantitative projections should not be extrapolated directly to countries with different health systems.

From a policy perspective, the concentration of the incidence increase in a small number of cancers implies that a targeted strategy would have disproportionate population-level impact: refining thyroid cancer screening to curb overdiagnosis, sustaining breast cancer early-detection and risk-based screening programs [24,31,32], optimizing prostate cancer PSA-testing pathways to balance benefit against overdetection, and—given the projected rise and poor prognosis of pancreatic cancer—prioritizing research investment in early-detection biomarkers and risk-stratified surveillance. The expanding five-year prevalence pool further argues for proactive planning of survivorship care, oncology workforce capacity, and long-term follow-up infrastructure.

## 5. Limitations

This study has several limitations. First and most importantly, the analysis is ecological: it uses aggregate national rates and therefore cannot support individual-level or causal inference, and correlations among cancer types may reflect shared secular trends rather than shared etiology (ecological fallacy) [14]. Second, the hierarchical regression is affected by compositional dependence and severe multicollinearity, so its coefficients are descriptive only. Third, the single-slope APC summarizes an average trend and does not capture the well-documented change-points (e.g., thyroid cancer around 2011–2016; the 2020 pandemic dip); joinpoint/AAPC trends are available in the official national statistics [5,6]. Fourth, the forecasts are extrapolative: they assume smooth continuation of historical trends and a stable age structure, and do not incorporate future changes in screening policy, diagnostic technology, or demographic shocks—so the projected values, while cross-validated on historical data, carry irreducible uncertainty. Fifth, we could not examine socioeconomic status, geographic region, or urban–rural differences, because the public KOSIS release used here is not stratified by these variables; subgroup and small-area analyses using record-level data are an important direction for future work. Sixth, ASIR estimates depend on the accuracy of census-based standard populations, and the attribution of the 2020 decline to screening disruption, though supported by national screening participation data [22], was not tested with linked screening records. Finally, five-year prevalence data were available only from 2007 onward, limiting the survivorship analysis relative to the full incidence series.

## 6. Conclusions

Korean cancer epidemiology has undergone a substantial transformation from 1999 to 2023, shifting from infection-related malignancies (stomach, liver) toward hormone-sensitive and screening-detectable cancers (thyroid, breast, prostate). Total incidence has nearly tripled and the five-year prevalence pool has exceeded one million persons. Inter-cancer correlation and hierarchical regression indicate that the overall increase is dominated by the co-movement of thyroid, breast, and prostate cancer, although—because these analyses are ecological and compositionally dependent—this should be read as a descriptive pattern rather than as identification of causal drivers. Cross-validated Holt–Winters forecasting, corroborated by an ARIMA benchmark and robust to the pandemic disruption, projects continued growth in total incidence through 2028, with prostate and pancreatic cancer rising most steeply. These findings provide an evidence base for targeted cancer control policy, survivorship-oriented healthcare planning, and future record-level epidemiological research in Korea.

## Figures and Tables

**Table 1:** Age-standardized incidence rates, annual case counts, and annual percent change for the top 10 cancers in Korea (1999–2023). Values were recomputed directly from the December 2025 revised registry release; endpoints therefore supersede those in the previous manuscript version.

Cancer	Total 1999	Total 2023	ASIR 1999	ASIR 2023	APC (%)
**All cancers**	**101,854**	**288,613**	**402.7**	**522.9**	+1.00 ***
Thyroid	3407	35,440	9.8	68.9	+7.56 ***
Lung	13,224	32,953	59.8	57.5	−0.13
Breast	5888	29,871	17.2	56.8	+5.03 ***
Stomach	20,900	28,943	86.0	51.4	−2.20 ***
Prostate	1454	22,640	7.4	39.2	+6.98 ***
Colon	4804	17,103	20.4	30.2	+1.46 *
Rectum	4979	15,507	20.4	28.5	+0.47
Liver	13,263	14,707	52.4	26.1	−2.90 ***
Pancreas	2614	9748	11.7	17.1	+1.65 ***
Other skin	1112	8297	5.0	14.3	+5.05 ***

ASIR: age-standardized incidence rate (per 100,000; 2020 Korean standard population). APC: annual percent change estimated from log-linear regression over 1999–2023. * *p* < 0.05; *** *p* < 0.001. Case counts for 1999 and ASIR endpoints were recomputed from the current registry release and differ from previously reported provisional values; APC estimates are unchanged.

**Table 2:** Hierarchical regression predicting overall age-standardized cancer incidence rate from cancer-specific rates.

Model	Variables	R^2^	ΔR^2^	Adj. R^2^	F	*p*-Value
Block 1	Lung + Stomach	0.449	—	0.399	8.98	0.0014
Block 2	+Thyroid, Breast, Prostate	0.997	0.547	0.995	1117.4	<0.0001
Block 3	All top 10 cancers	0.9997	0.003	0.9994	4927.4	<0.0001

ΔR^2^: change in R^2^ relative to the previous block. Cohen’s f^2^ for the Block-2 increment = 161.4.

**Table 3:** Variance inflation factors (VIFs) for the Block-3 regression predictors.

Predictor	VIF	Predictor	VIF
Thyroid	18.1	Colon	28.5
Lung	4.3	Rectum	14.0
Breast	263.3	Liver	263.2
Stomach	105.5	Pancreas	62.5
Prostate	99.5	Other skin	141.7

VIF > 10 conventionally indicates problematic multicollinearity. The extreme values confirm that individual regression coefficients cannot be interpreted as independent effects.

**Table 4:** Holt–Winters forecasts of cancer incidence for the top 10 cancers in Korea, 2024–2028.

Cancer	2023 (Obs.)	2024	2025	2026	2027	2028
Thyroid	35,440	36,132	36,685	37,128	37,483	37,766
Lung	32,953	34,102	35,019	35,932	36,840	37,744
Breast	29,871	31,583	32,872	34,155	35,432	36,702
Stomach	28,943	29,130	29,212	29,286	29,353	29,414
Prostate	22,640	24,244	25,844	27,435	29,018	30,593
Colon	17,103	17,403	17,649	17,884	18,108	18,321
Rectum	15,507	15,788	16,060	16,323	16,577	16,822
Liver	14,707	14,544	14,412	14,295	14,190	14,096
Pancreas	9748	10,173	10,572	10,968	11,362	11,754
Other skin	8297	8521	8915	9307	9697	10,085

Point forecasts from damped Holt–Winters exponential smoothing; 2023 values are observed. 95% prediction intervals are shown in Figure 6.

**Table 5:** Holt–Winters smoothing parameters and rolling-origin forecast accuracy, with ARIMA(1,1,1) benchmark.

Cancer	α	β	φ	HW RMSE	HW MAPE	ARIMA RMSE	ARIMA MAPE
Thyroid	1.00	0.74	0.80	5208	13.83%	5942	14.70%
Lung	0.33	0.33	0.99	919	2.38%	953	2.56%
Breast	0.36	0.36	0.99	1154	2.98%	1180	3.03%
Stomach	0.72	0.00	0.91	1739	4.83%	1470	3.91%
Prostate	1.00	0.33	0.99	968	5.08%	929	4.57%
Colon	0.95	0.00	0.95	990	5.43%	949	5.08%
Rectum	1.00	0.00	0.97	1097	4.62%	1081	4.55%
Liver	0.57	0.57	0.89	207	1.16%	213	1.00%
Pancreas	0.56	0.56	0.99	117	1.38%	189	2.07%
Other skin	0.41	0.41	0.99	320	3.91%	403	5.05%

α: level smoothing; β: trend smoothing; φ: damping. RMSE in cases; MAPE in percent; both from rolling-origin cross-validation (expanding window, one year ahead).

**Table 6:** Sensitivity analysis: APC (%) estimated over the full period versus with pandemic years (2020–2021) excluded.

Cancer	APC, Full Period	APC, Excl. 2020–2021	Δ (pp)
Thyroid	+7.56	+8.18	+0.62
Lung	−0.13	−0.09	+0.04
Breast	+5.03	+5.08	+0.05
Stomach	−2.20	−2.03	+0.17
Prostate	+6.98	+7.23	+0.25
Colon	+1.46	+1.76	+0.30
Rectum	+0.47	+0.62	+0.15
Liver	−2.90	−2.83	+0.07
Pancreas	+1.65	+1.66	+0.01
Other skin	+5.05	+5.25	+0.20

pp: percentage points. The small differences confirm that long-term trend estimates are not artefacts of the 2020 screening disruption.

## Data Availability

The analysis was based on aggregate raw data files derived from KOSIS (https://kosis.kr). All analyses were performed exclusively on pre-aggregated annual national statistics; no individual-level data were accessed. Aggregate national data are publicly available via KOSIS.

## Supplementary Materials

The following supporting information can be downloaded at: https://www.mdpi.com/article/10.3390/cancers18142341/s1, Table S1: Annual percent change (APC), 2023 incidence, and 2023 age-standardized incidence rate (ASIR) for all 61 cancer types in Korea, 1999–2023.
