# Supplementary material for: Epidemiological Trends, Inter-Cancer Correlations, and Incidence Projections for 61 Cancer Types in Korea, 1999–2028: A Nationwide Population-Based Study
Source: Cancers (Basel). 2026 Jul 20;18(14):2341. doi: 10.3390/cancers18142341 (PMC13406901; doi:10.3390/cancers18142341)
Supplement: Supplementary file 1 [file cancers-18-02341-s001.zip › cancers-4417297-supplementary.pdf]

Supplementary Materials

Table S1. Annual percent change (APC), 2023 incidence, and 2023 age-standardized incidence rate (ASIR) for all 61 cancer types in Korea, 1999–2023.

Cancer type (ICD-O)	2023 cases	ASIR 2023	APC (%)	p-value
Lip (C00)	54	0.1	-0.90	0.089
Tongue (C01–C02)	1,177	2.2	+2.45	<0.001
Mouth (C03–C06)	930	1.7	-0.22	0.691
Major salivary glands (C07–C08)	718	1.3	+2.31	<0.001
Tonsil (C09)	689	1.2	+3.65	<0.001
Oropharynx (C10)	207	0.4	+1.25	0.055
Nasopharynx (C11)	436	0.8	-0.94	<0.001
Hypopharynx (C12–C13)	596	1.0	-0.95	<0.001
Other/ill-defined oral (C14)	27	0.0	NA	NA
Esophagus (C15)	3,142	5.5	-1.75	<0.001
Stomach (C16)	28,943	51.4	-2.20	<0.001
Small intestine (C17)	1,279	2.3	+2.10	<0.001
Colon (C18)	17,103	30.2	+1.46	0.011
Rectum (C19–C20)	15,507	28.5	+0.47	0.226
Anus (C21)	365	0.7	+0.41	0.103
Liver (C22)	14,707	26.1	-2.90	<0.001
Gallbladder & biliary tract (C23–C24)	7,997	13.8	-0.05	0.698
Pancreas (C25)	9,748	17.1	+1.65	<0.001
Nasal cavity & sinuses (C30–C31)	501	0.9	-0.41	0.017
Larynx (C32)	1,254	2.2	-3.18	<0.001
Lung (C33–C34)	32,953	57.5	-0.13	0.061
Other thoracic (C37–C38)	1,298	2.4	+4.58	<0.001
Bone & articular cartilage (C40–C41)	446	0.8	-0.45	0.105
Malignant melanoma of skin (C43)	713	1.3	+1.68	<0.001
Other skin (C44)	8,297	14.3	+5.05	<0.001
Mesothelioma (C45)	174	0.3	+2.50	<0.001
Kaposi sarcoma (C46)	83	0.1	NA	NA
Connective & soft tissue (C47+C49)	1,311	2.4	+1.72	<0.001
Breast (C50)	29,871	56.8	+5.03	<0.001
Vulva (C51)	230	0.4	+2.25	<0.001
Vagina (C52)	84	0.2	-0.74	0.165
Cervix uteri (C53)	3,144	5.9	-3.81	<0.001
Corpus uteri (C54)	4,037	7.6	+5.03	<0.001
Uterus, unspecified (C55)	163	0.3	-2.96	<0.001
Ovary (C56)	3,299	6.2	+1.72	<0.001
Other female genital (C57)	269	0.5	+6.54	<0.001
Placenta (C58)	15	0.0	NA	NA
Penis (C60)	68	0.1	-3.25	<0.001
Prostate (C61)	22,640	39.2	+6.98	<0.001
Testis (C62)	315	0.6	+5.39	<0.001
Other male genital (C63)	130	0.2	+3.97	<0.001
Kidney (C64)	7,367	13.5	+3.93	<0.001

Cancer type (ICD-O)	2023 cases	ASIR 2023	APC (%)	p-value
Renal pelvis (C65)	782	1.4	+3.55	<0.001
Ureter (C66)	834	1.4	+3.55	<0.001
Bladder (C67)	5,545	9.6	-0.32	0.004
Other urinary (C68)	227	0.4	+3.21	<0.001
Eye (C69)	139	0.3	-0.09	0.872
Brain & CNS (C70–C72)	2,080	3.9	+0.01	0.914
Thyroid (C73)	35,440	68.9	+7.56	<0.001
Adrenal gland (C74)	444	0.8	+3.29	<0.001
Other endocrine (C75)	954	1.8	+5.36	<0.001
Hodgkin lymphoma (C81)	356	0.7	+2.84	<0.001
Non-Hodgkin lymphoma (C82–C86, C96)	6,109	11.1	+2.28	<0.001
Immunoproliferative disease (C88)	1,560	2.9	+9.30	<0.001
Multiple myeloma (C90)	2,099	3.7	+2.88	<0.001
Lymphoid leukemia (C91)	1,076	2.1	+2.50	<0.001
Myeloid leukemia (C92–C94)	2,889	5.3	+1.80	<0.001
Leukemia, unspecified (C95)	253	0.4	-5.03	<0.001
Myeloproliferative disorders	1,763	3.2	+7.94	<0.001
Myelodysplastic syndromes	1,715	3.0	+6.79	<0.001
Other & unspecified (C00–C96)	2,061	3.6	-4.34	<0.001

ASIR: age-standardized incidence rate (per 100,000; 2020 Korean standard population). APC estimated from log-linear regression of ASIR on year (1999–2023). Cancer types with any zero/missing ASIR are shown as NA. Data source: Korea Central Cancer Registry via KOSIS (Table ID DT_117N_A00025).
